# MAP kinase meets mitosis: A role for Raf Kinase Inhibitory Protein in spindle checkpoint regulation

**DOI:** 10.1186/1747-1028-2-1

**Published:** 2007-01-10

**Authors:** Marsha Rich Rosner

**Affiliations:** 1 Ben May Department for Cancer Research, University of Chicago, Chicago, IL 60637, USA

## Abstract

Raf Kinase Inhibitory Protein (RKIP) is an evolutionarily conserved protein that functions as a modulator of signaling by the MAP kinase cascade. Implicated as a metastasis suppressor, Raf Kinase Inhibitory Protein depletion correlates with poor prognosis for breast, prostate and melanoma tumors but the mechanism is unknown. Recent evidence indicates that Raf Kinase Inhibitory Protein regulates the mitotic spindle assembly checkpoint by controlling Aurora B Kinase activity, and the mechanism involves Raf/MEK/ERK signaling. In contrast to elevated MAP kinase signaling during the G1, S or G2 phases of the cell cycle that activates checkpoints and induces arrest or senescence, loss of RKIP during M phase leads to bypass of the spindle assembly checkpoint and the generation of chromosomal abnormalities. These results reveal a role for Raf Kinase Inhibitory Protein and the MAP kinase cascade in ensuring the fidelity of chromosome segregation prior to cell division. Furthermore, these data highlight the need for precise titration of the MAP kinase signal to ensure the integrity of the spindle assembly process and provide a mechanism for generating genomic instability in tumors. Finally, these results raise the possibility that RKIP status in tumors could influence the efficacy of treatments such as poisons that stimulate the Aurora B-dependent spindle assembly checkpoint.

## Background

Signaling cascades, the means by which cells translate external stimuli into discrete physiological endpoints such as cell growth, must ensure high fidelity and specificity in order to maintain cellular integrity. One of the key steps regulating cell cycle progression occurs during mitosis when proper kinetochore attachment to chromosomes enables equal and ordered separation of chromosomal DNA to daughter cells. This process, regulated by the spindle assembly checkpoint, is under exquisite control to ensure that the cell corrects mistakes prior to cell cycle exit and cell division. Many of the key regulators of this checkpoint have been identified including DNA attachment proteins, kinesins that drive dynamic changes, checkpoint proteins that arrest the cycle, and proteins involved in proteosome activity that drive the process forward. Loss of any of these proteins is deleterious to the progression of the cell cycle and leads to catastrophic consequences if not corrected.

One of the mechanisms by which the cell ensures signaling fidelity involves modulators of signaling cascades such as scaffold proteins that potentiate or inhibit the output. Depletion of these proteins would not compromise the cell acutely but could lead to slow accumulation of chromosomal abnormalities culminating in mutation and disease. One such modulator, Raf Kinase Inhibitory Protein (RKIP), was recently shown to function via MAP kinase as a regulator of the spindle assembly checkpoint [1].

RKIP, also termed phosphatidylethanonolamine binding protein (PEBP), is an evolutionarily conserved protein that regulates growth and differentiation in a variety of species [2,3]. In mammalian cells, RKIP functions as an inhibitor of the MAP kinase signaling module comprised of Raf-1/MEK/ERK1,2 [4]. RKIP inhibits Raf-1 activation by preventing phosphorylation of key regulatory sites on Raf-1 [5]. Upon growth factor stimulation of cells, RKIP is phosphorylated at S153 by protein kinase C (PKC) causing its dissociation from Raf-1[6]. The released, phosphorylated RKIP binds and inhibits GRK2, potentiating G protein-coupled receptor (GPCR) signaling [7]. Thus, RKIP functions as an environmental sensor or "switch" that flips from down regulating the amplitude and dose response of Raf-1-mediated ERK activation and resultant DNA synthesis to ameliorating GPCR down regulation [2,5].

As a regulator of MAP kinase signaling, RKIP has been implicated in cancer progression. RKIP expression is reduced in prostate, melanoma and breast cancer, and this decrease correlates with the extent of metastatic disease [8,9]. In a xenograft mouse model for prostate cancer, exogenous RKIP expression suppresses invasion and metastasis, and this reduction correlates with Raf-1 inhibition [10]. RKIP also potentiates apoptosis induced by chemotherapeutic agents, and this has been attributed in part to its reported inhibition of TNF-α-activated IKKβ in the NFkB cell survival pathway [11,12]. Since RKIP suppresses metastatic progression in a variety of cancers, it is likely that RKIP affects a fundamental step in the process rather than targeting tumor-specific mechanisms of invasion or colonization.

## Discussion

Recently, a new role for RKIP as a regulator of mitotic progression during the cell cycle was reported [1]. Initially, the phosphorylated form of RKIP (pS153) indicative of Raf-1 activation was detected in association with centrosomes and prophase/prometaphase kinetochores in a number of cells and tissues including prostate cancer cells, brain hippocampus, and head and neck tumor tissues. Analysis of several rat and human cell types including HeLa and rat H19-7 hippocampal cells revealed a decrease in mitotic index upon RKIP depletion and, specifically, of cells in metaphase. Cell growth rates and apoptotic rates were unaffected, but a decrease in the time of mitotic traversal from nuclear envelope breakdown to anaphase accounted for the observed phenotype. Furthermore, RKIP-depleted cells overrode the spindle checkpoint triggered by loss of spindle tension after Taxol but not acute nocodazole treatment, resulting in an increase in Taxol-induced chromosomal defects.

These phenotypes, including the bypass of the spindle checkpoint, result from the regulation of Aurora B kinase by RKIP. Aurora B [13] is an evolutionarily conserved kinase that has been implicated in chromosomal alignment, cytokinesis, and spindle checkpoints. In complex with other "chromosomal passenger" proteins, Aurora B accumulates at inner centromeres during prometaphase and controls the interactions of microtubules with kinetochores. The spindle checkpoint delays chromosome segregation in response to problems with spindle attachment or tension at the kinetochores [14]. This checkpoint is not only triggered by spindle damage but also plays a role in the initiation of anaphase in every cell. Aurora B kinase specifically regulates activation of the spindle checkpoint in response to loss of tension due to poisons such as Taxol, [15,16]. A decrease in Aurora B localization and kinase activity at the kinetochore alters the integrity of the spindle assembly checkpoint, the same phenotype observed in RKIP-depleted cells exposed to Taxol. Consistent with this observation, RKIP depletion causes inhibition of Aurora B kinase activity and decreased detection of phosphorylated Aurora B and its substrate CENP-A at kinetochores.

The mitotic defects elicited by RKIP depletion were due, at least in part, to elevated MAP kinase activity. Activated Raf-1 was localized on early mitotic kinetochores, activation of the MAP kinase cascade mimicked effects of RKIP depletion, and inhibition of ERK1,2 signaling by either drugs, dominant-negative MEK, or siRNA for Raf-1 rescued defects caused by RKIP loss. The specific mitotic target of MAP kinase action has not yet been identified. The passenger protein complex consisting of Aurora B, INCENP, Survivin and Borealin/Dasra B is required for maintaining the integrity of mitotic regulation, including phosphorylation of histone H3 and its variant CENP-A, spindle assembly, chromatin-induced stabilization of microtubules, and mitotic arrest in response to microtubule poisons such as Taxol [17]. The passenger proteins stabilize Aurora B localization to the inner centromere and are required for kinetochore-associated Aurora B kinase activity. Although Aurora B is not a direct substrate of MAP kinase, it is possible that MAP kinase regulates the translocation of Aurora B to kinetochores or Aurora B kinase activity by phosphorylation of the passenger proteins. Taken together, the results of this study[1] demonstrate that loss of RKIP, through hyperactivation of the Raf/MEK/ERK1,2 signaling cascade, regulates the mitotic checkpoint via inhibition of Aurora B kinase.

The Raf/MAPK signaling cascade has been implicated previously in regulation of cell cycle checkpoints during G1, S and G2. However, the role of the MAPK pathway during the G2/M phase of the mammalian mitotic cell cycle has been a subject of considerable controversy. Several studies have shown that excessive activation of Raf and MAP kinase in G1 leads to upregulation of cyclin-dependent kinase inhibitors, culminating in cell arrest or senescence [18,19]. Depletion of the dual-specific tyrosine phosphatase VHR upregulates p21 and causes arrest or senescence at the G1-S and G2-M stages of the cell cycle [20]. VHR down regulates both ERK1,2 and JNK, and inhibition of both of these MAP kinase subfamilies partially rescues the cell arrest phenotype. Raf-1-activated MEK1 and ERK1c, an ERK variant, promote mitotic Golgi fragmentation and G2 progression [21,22]. Oncogenic H-Ras expression in thyroid cells leads to bypass of the G2 DNA damage checkpoint in a MEK/ERK-dependent manner, but activation of the B-Raf pathway alone is insufficient for the checkpoint override [23]. Very recently, Shapiro and coworkers showed that cells synchronized at the G1-S boundary and then stimulated by EGF or TPA induce p21 and transient arrest at the G2-M boundary in a MEK/ERK-dependent fashion [24]. The apparent contradictory ability of ERK to promote G2/M arrest but also potentiate bypass of the G2 checkpoint presumably reflects differences in stimuli and cell type. Although more work needs to be done to sort out the specific role of MAP kinase in G2, these results indicate that the Raf/MEK/ERK cascade plays a key regulatory role in the G1-S and G2-M stages of the cell cycle. Given that RKIP influences the amplitude of ERK signaling, it is likely that RKIP may play a regulatory role in the G1-S and G2 stages of the cell cycle as well as in mitosis.

How does this new finding about RKIP regulation of the spindle checkpoint relate to our understanding of MAPK and mitosis? Although many of the fundamental players in mitotic regulation of eukaryotes were discovered using Xenopus eggs as a model system, the function of MAPK in this system is distinct from that in mammalian cells. In Xenopus egg extracts, MAPK is not required for mitotic entry or exit, and MAPK activation promotes cell cycle arrest [25,26]. By contrast, in mammalian cells, the Raf-1/ERK1,2 cascade may influence entry into mitosis and functions to bypass spindle checkpoint arrest [1,27,28]. Acute activation of either B-Raf or the Raf kinase domain during G2 leads to a decrease in the mitotic index and at least partial override of the spindle checkpoint [1,23]. Consistent with this role at the metaphase/anaphase boundary, active Raf-1 and ERK1,2 are associated with kinetochores, and active ERK has been localized to spindle poles from prometaphase to anaphase and with the midbody at later stages of mitosis [1,29,30]. However, it is possible that RKIP and ERK target an earlier step in the mitotic process. As a regulator of the spindle checkpoint, RKIP functions as a negative modulator that controls the amplitude and dose response of Raf-1 kinase activity rather than the absolute on or off state. Interestingly, excessive MAP kinase activity drives cell arrest or senescence at the G1-S and G2-M boundaries, but causes cells to bypass the spindle checkpoint in the absence of normal RKIP expression.

## Conclusion

The regulation of the mitotic spindle checkpoint by RKIP provides a potential explanation for some of its growth and tumor-regulating functions. Treatment of cells with chemotherapeutic agents such as Taxol can enhance RKIP expression in the arrested cells [11]. These results suggest that this enhancement may be caused to some extent by the normal increase in RKIP expression that occurs during mitosis. Similarly, it has been shown that RKIP can potentiate the extent of apoptotic death induced by chemotherapy [11]. If RKIP promotes arrest or apoptosis due to the mitotic checkpoint, then higher levels of RKIP should increase cell death. Conversely, depletion of RKIP should lead to slippage of cells through the checkpoint resulting in fewer arrested or apoptotic cells and an increase in aneuploidy depending upon the specific cell type. In fact, expression of oncogenic Ras, an upstream activator of Raf-1, has been shown to promote chromosome instability via ERK [31]. The possibility that RKIP depletion promotes genomic instability, similar to spindle checkpoint proteins, needs to be tested directly in a mouse model.

Partial suppression of the spindle checkpoint rather than its total elimination is more likely to lead to cancer given that complete inactivation could result in cell death [14]. RKIP depletion causes a partial suppression of the spindle checkpoint in cells exposed to low levels of Taxol. Higher Taxol levels induce dramatic chromosomal abnormalities and eventually catastrophic cell death. Interestingly, RKIP itself does not trigger cell death unless overexpressed or mutated to block dissociation from Raf-1. Conversely, loss of endogenous RKIP or enhanced Raf kinase activation induces a spindle checkpoint defect that enables cells to escape Taxol-induced arrest more easily. Cells either proceed through division or die dependent on the dose, suggesting that RKIP levels in cancer cells can influence the Taxol regimen needed for toxicity. These data indicate that Raf-1 kinase activity must be tightly regulated during mitosis, and RKIP plays a key role in modulating this activity. Cells lacking RKIP should display an increase in chromosomal abnormalities and genetic changes when under oncogenic or toxic stress. Future therapeutic strategies for cancer treatment should be able to take advantage of the RKIP status of tumor cells in order to selectively potentiate cell death.

## List of abbreviations

RKIP, Raf Kinase Inhibitory Protein; MAP kinase, mitogen activated protein kinase; ERK, extracellular signal regulated kinase; MEK, MAP/ERK kinase; PKC, protein kinase C

## Competing interests

The author(s) declare that they have no competing interests.
